# The Effect of Creative Tasks on Electrocardiogram: Using Linear and Nonlinear Features in Combination with Classification Approaches

**Published:** 2017-01

**Authors:** Sahar Zakeri, Ataollah Abbasi, Ateke Goshvarpour

**Affiliations:** 1 M.Sc., Computational Neuroscience Laboratory, Department of Biomedical Engineering, Faculty of Electrical Engineering, Sahand University of Technology, Tabriz, Iran.; 2 Associate Professor, Computational Neuroscience Laboratory, Department of Biomedical Engineering, Faculty of Electrical Engineering, Sahand University of Technology, Tabriz, Iran.; 3 Ph.D. Student, Computational, Neuroscience Laboratory, Department of Biomedical Engineering, Faculty of Electrical Engineering, Sahand University of Technology, Tabriz, Iran.

**Keywords:** *Adaptive Neuro-Fuzzy Inference System*, *Creativity Level*, *Electrocardiogram*, *Features Extraction*, *Support Vector Machine*, *Torrance Tests of Creative Thinking*

## Abstract

**Objective: **Interest in the subject of creativity and its impacts on human life is growing extensively. However, only a few surveys pay attention to the relation between creativity and physiological changes. This paper presents a novel approach to distinguish between creativity states from electrocardiogram signals. Nineteen linear and nonlinear features of the cardiac signal were extracted to detect creativity states.

**Method:** ECG signals of 52 participants were recorded while doing three tasks of Torrance Tests of Creative Thinking (TTCT/ figural B). To remove artifacts, notch filter 50 Hz and Chebyshev II were applied. According to TTCT scores, participants were categorized into the high and low creativity groups: Participants with scores higher than 70 were assigned into the high creativity group and those with scores less than 30 were considered as low creativity group. Some linear and nonlinear features were extracted from the ECGs. Then, Support Vector Machine (SVM) and Adaptive Neuro-Fuzzy Inference System (ANFIS) were used to classify the groups.

**Results:** Applying the Wilcoxon test, significant differences were observed between rest and each three tasks of creativity. However, better discrimination was performed between rest and the first task. In addition, there were no statistical differences between the second and third task of the test. The results indicated that the SVM effectively detects all the three tasks from the rest, particularly the task 1 and reached the maximum accuracy of 99.63% in the linear analysis. In addition, the high creative group was separated from the low creative group with the accuracy of 98.41%.

**Conclusion**: the combination of SVM classifier with linear features can be useful to show the relation between creativity and physiological changes.

Nowadays, ECG is an important tool in many scientific fields (1). Han and Wolf discovered the relationship between the nervous and cardiovascular system in 1963 (2). As the heart muscle generates ECG signals, information from normal and pathological states of heart can be understood from the ECG signals (3, 4). Over the past few decades, many computational tools and methods for analyzing these signals have been proposed. These tools automatically analyze the electricity of the heart and reveal the cardiac anomalies. Many researchers are interested in detecting cardiac behavior like stress (5), anger and fear (6) or psychiatric diseases (7) by applying ECG signals processing. 

Historic review on creativity suggests that in recent decades there has been great interest towards creativity education. In 1956, Torrance designed various types of tests to determine the creativity that people still used in many studies (8, 9).

Brainstorming is performed to nurture creativity exercises and in this technique fluency, flexibility and innovation are used (8-12).

Creative thinking is closely related to the nervous system, which consists of the sympathetic nervous system (SNS) and the parasympathetic nervous system (PNS). The SNS activities increase heart rate and ECG (Nguyen, Zeng, 2014). Therefore, SNS becomes dominant and causes biological and psychological changes to adjust the body to the creative situation. On the other hand, the critical signals of the body help diagnose cognitive behavior. In 2006, Ghacibeh et al. (13) found that any stimulation of the vagus nerve (sympathetic system) is associated with raised heart rate, causing injury test and thus reducing the level of individual creativity. Previously, electroencephalography (EEG) signals were used in the detection of creativity states (14). Although these studies have been done statistically, detailed analysis has not been yet done based on feature extraction. In addition, the role of physiological signals such as ECG has not been studied. For the first time, in this paper, not only the creative effects on psychological parameters with ECG analysis is checked, but also an algorithm is suggested to classify creativity states.

The aim of this study was to separate different aspects of creativity with ECG signals. To this end, time domain and frequency domain characteristics of linear features and nonlinear features like Renyi’s entropy and fractal dimension were extracted from ECG signals in rest and creativity states. Then all features were applied on two classifiers. Figure 1 demonstrates the research schematically.

## Materials and Methods

***Data Collection***

In this study, to assess the student’s creative thinking, we used Torrance Test of Creative Thinking (TTCT) Form B (figural). The test of high discrimination for evaluating creativity has cognitive components, mainly in the form of assignments, practicable creativity and creative problem-solving techniques. The ECG signals were collected of 52 students of biomedical engineering, material engineering and control engineering (26 female and 26 male; 19-24 years). The participants were asked to get enough sleep and not to drink coffee for five hours. All tasks were explained to the participants before recording. They sat on comfortable chairs, and electrodes were connected to their wrists. They were present in the lab half-hour before. The observer had a conversation with the participant to relax them. ECG signals were recorded from lead II, 2-minutes of rest states and 30 minutes in creativity states, while the TTCT was done. Sampling frequency was 1000 Hz. ECG signals were recorded in the Computational Neuroscience Laboratory of Sahand University of Technology.

***Pre-Processing***

ECG recordings are combined with high-frequency noises such as crosstalk, Electromyography noise and other equipment (1). All signals were pre-processed with Notch filter 50Hz to remove power line noise. Chebyshev 2 with an order of 4 (low cut-off frequency of 0.3 Hz and high cut-off frequency 250Hz) was also used to eliminate ambient noises. The first 0.6 seconds of ECG signal were removed from all records. Window sampling was five-seconds (3). The first two-minutes of each 10-minute ECG signal was considered in the analysis. 

**Linear and Nonlinear Features**

***Time domain***

 Simple time domain variables that can be calculated include the mean Normal to Normal (NN) interval, the mean heart rate and the difference between the longest and shortest NN interval. The simplest variable to calculate is the standard deviation of the NN interval (SDNN), and the square root of the variance. Other commonly used statistical variables calculated from segments of the total monitoring period include the NN50 (the normal-to-normal intervals that more than 50-second) and pNN50 (the number of the NN50 divided by 100). RMSD, the root mean square deviation and median, minimum, the maximum were other features in time domain (15).

***Frequency domain***

The power spectral density (PSD) analysis provides the basic information of how power, and therefore the variance, distribute as a role of frequency. The different frequency bands are as follows: Ultra-low frequency component (ULF) below 0.003 Hz, a very low-frequency component (VLF) from 0.003 to 0.04 Hz, a low-frequency component (LF) from 0.04 to 0.15 Hz, and high-frequency component (HF) 0.15 to 0.4 Hz (16). In addition, the ratio LF to HF is a frequency domain feature that indicates the sympathetic and parasympathetic balance of the heart.

***Fractal Dimension***

A fractal is a set of points that when looked at smaller scales, resembles the whole set. An essential characteristic of a fractal is self-similarity (15). This means that its details at a certain scale are similar, but not necessarily identical to those of the structure seen at larger or smaller scales. The concept of fractal dimension (FD) that refers to a non-integer or fractional dimension originates from fractal geometry. The FD emerges to provide a measure of how much space an object occupies between Euclidean dimensions. The FD of a waveform represents a powerful tool for transient detection. This feature has been used in the analysis of ECG to identify and distinguish specific states of physiological function. Several algorithms are available to determine the FD of the waveform, among others is the algorithms proposed by Higuchi and Katz (15). According to the method of Katz, the FD of a curve can be defined as follows:


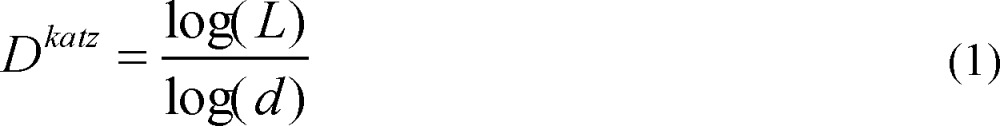


Where “L” is the total length of the curve or sum of distances between successive points, and d is the diameter estimated as the distance between the first point of the sequence and the most distal point of the sequence. Mathematically, d can be expressed as:


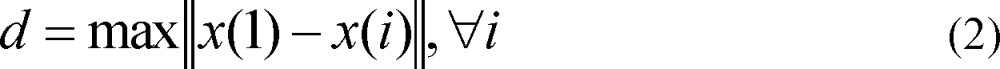


Considering the distance between each point of the sequence and the first, point i is the one that maximizes the distance with respect to the first point. The FD compares the actual number of units that compose a curve with the minimum number of units needed to reproduce a pattern of the same spatial extent. FDs calculated in this fashion depend on the measurement units used. If the units are different, then so are the FDs. Katz approach solves this problem by creating a general unit or yardstick: The average step or average distance between successive points, a. Normalizing the distances, Dkatz is then given by:


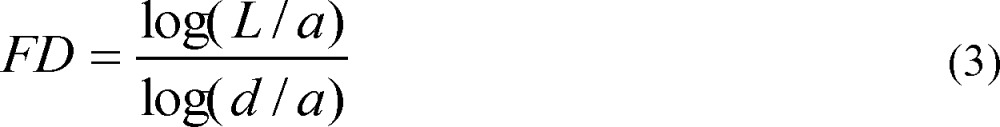


***Entropy***

Entropy refers to system randomness, regularity and predictability and allows systems to be quantified by rate of information loss or generation. Different types of entropies have been introduced (17). Renyi entropy is defined as:


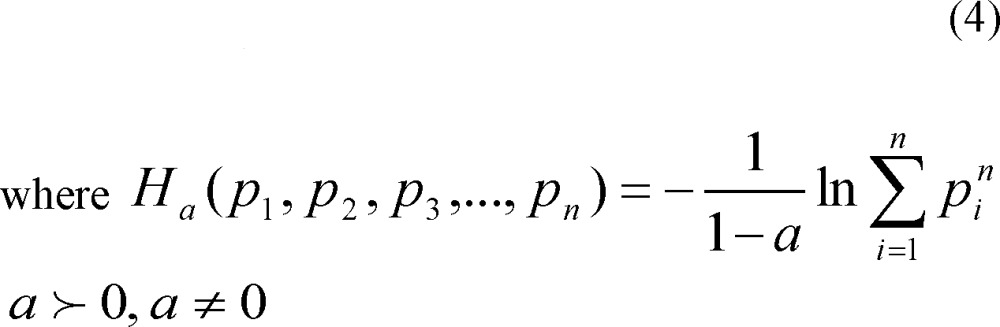


 “p_i” is the probability of a given symbol and p (1) consider 1, so p (i) = P (i-1)2+1. Entropy provides a lower bound for the compression that can be achieved by the data representation (coding) compression step. If a→1, equation (4) tends to the Shannon's measure of entropy (18). Another entropy called “the Non-normalized Shannon entropy” was also extracted. This type of entropy results from wavelet coefficients. Therefore, wavelet analysis based on wavelet entropy can measure the complexity of transient signals in both time and frequency domains. Shannon entropy can be used as follows:


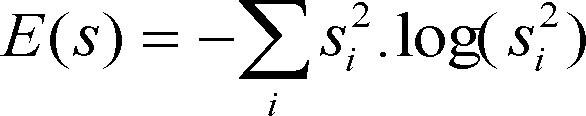


Where “s” is the signal and (si) is the coefficients of “s” in an orthonormal basis (19). Other types of the wavelet entropy such as Log energy entropy are calculated after taking logarithms from wavelet coefficients. Also, “Norm entropy” is extracted from the data in the norm forms (the norm is a normalized form that it take place between minimum and maximum values). In general, all these kinds of entropies were applied to identify the complexity of the signal in various ways. In physics, the word entropy has important physical implications as amount of "disorder" of a system. In mathematics, a more abstract definition is used. 

***Classifier***

***A.   Support vector machine***

Support Vector Machine (SVM) approach is used for the automatic classification of electrocardiogram (ECG) beats (20). Since an SVM is known to have the advantage of offering the solid performance of classification with even smaller learning data, the proposed algorithm, with rather small learning data, could prove better performance than other classifiers. The SVM classification whit Kernel function was used in this paper to classify ECG signal data (21). The Kernel is a mapping done to the training data to improve its resemblance to a linearly separable set of data. This mapping consists of increasing the dimensionality of the data and is done efficiently using a kernel function (20). In the SVM learning, the (Gaussian) radial basis function kernel, or RBF kernel, is a popular kernel function used in various kernelized learning algorithms. These parameters have been set using Lib-SVM toolbox.

***B.   Adaptive Neuro-Fuzzy Inference System***

The Adaptive Neuro-Fuzzy Inference System (ANFIS) classifier is used to obtain an improved diagnostic accuracy in the ECG signals (22) on creativity and normal states. The proposed ANFIS model combined the Neural Network adaptive capabilities and the fuzzy Inference System. In this study, the network used sub-clustering and hybrid model consisting of six rules and three hidden layers. Gaussian membership function was selected. A straightforward fuzzification is usually based on minimum and maximum operations, because in this case more properties of traditional mathematics can be extended to the fuzzy case. Thus, in this article, the minimum operation was selected for fuzzification. In addition, a useful defuzzification technique, center of gravity (23), was selected.

***TTCT Scores***

The ECG signals were recorded during the TTCT-Form B (figural type). TTCT contains three separate tasks (1- picture construction, 2- picture completion, 3- lines). Each task takes about 10 minutes; therefore, the entire TTCT lasted about 30 minutes. Task 1 of TTCT has two parameters for scoring (originality and elaboration), but task 2 and task 3 have four parameters for scoring (originality, elaboration, flexibility, and fluency). Normalization and z-transform were applied on the TTCT Scores. Then all scores were in the range from zero to 100 point. Therefore, two groups of participants with a high creative level (grades over 70) and with a low creativity (grades under 30) were isolated. Both groups were given the SVM classifier.

## Results

***Linear Analysis***

Results of time domain, frequency domain and nonlinear features are presented in Table 1. 

Table 1 demonstrates the average of the time domain parameters of rest and creativity tasks in ECG signals. In this work, 8-time domain features were found to be significant (p < 0.05). They are mean, SDNN, RMSD, minimum, maximum, median, NN50 and pNN50 of ECG (Figure 2). In general, the behavioral pattern of ECG parameters for the first task is different from other states (Figure 2). To determine the difference between the parameters, we used Wilcoxon test on data; the p value of its domain is listed in Table 1.

The significant features like median, minimum, SDNN and RMSD had lower values for the rest states compared to the creativity states. The next significant parameter was mean of ECG.

Significant features were obtained in the frequency domain analysis.

Similar to the time domain analysis, the behavioral pattern of ECG parameters for the first task was different from other states in frequency domain analysis (Figure 3).

The p-value of Wilcoxon test on frequency domain features is presented in Table 1.

Most of the frequency domain features were significantly different between all tasks and rest, except between rest states and the third task of creativity states (Table 1).

**Table1:** P Values of the Fourteen Linear Features in Time Domain and Frequency Domain extracted from ECG Signals between Rest Condition and Each Creative Task and between Each Pair of Creative Task

**Features**	**Rest vs. ** **Task 1**	**Rest vs. ** **Task 2**	**Rest vs. ** **Task 3**	**Task 1 vs. ** **Task 2**	**Task 1 vs. ** **Task 3**	**Task 2 vs. ** **Task 3**
Mean	<0.0001*	<0.0001*	<0.0001*	0.0559	0.9480	<0.0001*
RMSD	<0.0001*	0.0098*	0.1580	<0.0001*	<0.0001*	0.1885
SDNN	<0.0001*	<0.0001*	0.1580	<0.0001*	<0.0001*	0.1887
Minimum	<0.0001*	<0.0001*	<0.0001*	<0.0001*	<0.0001*	<0.0001*
Maximum	<0.0001*	0*	0.2121	0*	<0.0001*	0*
Median	<0.0001*	<0.0001*	<0.0001*	<0.0001*	<0.0001*	<0.0001*
NN50	0.0709	0.6657	0.0412*	0.1834	0.7497	0.1071
pNN50	0.0709	0.6657	0.0412*	0.1834	0.7497	0.1071
Total power	<0.0001*	0.0062	0.2055	<0.0001*	<0.0001*	0.1088
ULF	<0.0001*	<0.0001*	0.2695	0.0377	<0.0001*	<0.0001*
VLF	<0.0001*	<0.0001*	0.9109	<0.0001*	<0.0001*	<0.0001*
LF	<0.0001*	<0.0001*	0.2912	<0.0001*	<0.0001*	<0.0001*
HF	<0.0001*	<0.0001*	0.1862	<0.0001*	<0.0001*	<0.0001*
LF/HF	<0.0001*	0.4304	<0.0001*	<0.0001*	0.0862	<0.0001*

* indicated p<0.05.

**Table2:** P Value of the Nonlinear Features between Two States of Rest and Creativity Tasks Extracted from ECG Signals

	**FD**	**Renyi**	**Wavelet Shannon**	**Wavelet Norm**	**Wavelet Log**
Rest vs. task 1	<0.0001*	0.0100*	<0.0001*	<0.0001*	<0.0001*
Rest vs. task 2	<0.0001*	0.3065	0.0185*	<0.0001*	0.7156
Rest vs. task 3	<0.0001*	0.2435	0.9889	0.0048*	0.8355
Task 1 vs. Task 2	0.1412	0.1104	<0.0001*	<0.0001*	0.0019*
Task 1 vs. Task 3	0.0610	0.1918	<0.0001*	<0.0001*	<0.0001*
Task 2 vs. Task 3	<0.0001*	0.8225	0.0262*	0.5415	0.5824

* indicated p<0.05.

**Table3:** a) Results of SVM Classifier on 14 Linear Features Extracted from ECG Signals to Discriminate between Two Different States

	**Rest vs. ** **Task 1**	**Rest vs. ** **Task 2**	**Rest vs. ** **Task 3**	**Task 1 vs. ** **Task 2**	**Task 1 vs. ** **Task 3**	**Task 2 vs. ** **Task 3**
% accuracy rate	97.57	99.63	97.51	99.23	97.66	98.91
% Sensitivity	97.91	100	96.71	98.43	97.08	97.49
% Specificity	97.23	99.25	98.15	100	98.10	100
Likelihood ratio positive = sensitivity / (1 − specificity)	35.35	>100	52.28	>100	51.10	>100
Likelihood ratio negative = (1 − sensitivity) / specificity	0.02	0	0.04	0.02	0.03	0.03
% Error rate	2.43	0.37	3.35	0.767	2.34	1.09
**b) Results of SVM Classifier on 5 Nonlinear Features Extracted from ECG Signals to Discriminate between Two Different States**						
% Accuracy rate	86.06	79.71	82.36	72.76	72.43	58.34
% Sensitivity	86.11	77.63	84.92	78.83	78.33	69.61
% Specificity	86.02	82.05	80.38	66.77	67.78	49.11
Likelihood ratio positive = sensitivity / (1− specificity)	6.16	4.33	4.33	2.37	2.43	1.37
Likelihood ratio negative = (1−sensitivity) / specificity	0.16	0.27	0.19	0.32	0.32	0.62
% Error rate	13.94	20.29	17.64	27.24	27.57	41.66

**Table4:** ANFIS Classification Performances on Linear and Nonlinear Features of ECG Signals between Two states

**Linear features of ECG signals**						
	**Rest vs. ** **Task 1**	**Rest vs. ** **Task 2**	**Rest vs. ** **Task 3**	**Task 1 vs. ** **Task 2**	**Task 1 vs. ** **Task 3**	**Task 2 vs. ** **Task 3**
%Error rate	2.77	37.10	5.96	15.98	31.23	30.38
Sensitivity	91.61	68.39	87.78	65.34	69.57	67.12
Specificity	67.79	57.53	60.62	56.25	50.75	59.95
**Nonlinear features of ECG signals**						
%Error rate	48.06	43.09	45.66	37.87	43.14	41.10
Sensitivity	40.11	45.47	42.37	51.49	45.68	47.36
Specificity	38.85	34.01	32.13	52.21	36.40	38.99

ULF, VLF, LF and HF increase from rest to task 1 and decrease from task 1 to task 2 and task 3. However, the ratio of LF to HF was increased from task 1 to task 2, and in the end it was less than the initial value.

***Nonlinear Analysis***

We calculated five nonlinear features on ECG signals. Figure 2 demonstrates the changes of all five nonlinear features associated with the TTCT activity changes. Based on Figure 4 and Table 2, FD, entropy wavelet Shannon and norm showed a significant difference between most groups (rest and creativity states). The value of Renyi entropy, wavelet norm and wavelet Shannon were increased from rest to task 1, but entropy wavelet log was decreased in this step. FD had a higher value in rest states rather than that of creativity states. The P value of these nonlinear features is presented in Table 2.

***SVM Classifier***

SVM classification applying Kernel function was used in this paper to classify ECG signal; 70% of the data were applied on the trained group and 30% on the test group. Table 3.a and 3.b present SVM classification results. In linear features, SVM classification showed less error 0.767% between two classes of task 1 and task 2 (Table 3.a). Higher value of accuracy (99.63%) and sensitivity (100%) was achieved between rest and task 2. The SVM classification had no appropriate accuracy and sensitivity to classify states 3 ​​and 4. To validate the classifier’s output, the mean 20 times run of the classification has been reported.

Athwart linear features, in nonlinear features SVM classification gives less error (13.94%) between two classes of rest and task 1 (Table 3.b) and high value of accuracy (86.06%) and sensitivity (86.11%) was observed in this class. Based on Table 3, it was concluded that linear features have a high value of accuracy and sensitivity in all the classifieds; they were higher than 96%, but nonlinear features had less accuracy and sensitivity and they were less than 86%.

***ANFIS Classifier***

In this study, 70% of the data were used in the trained group and the rest in the test group (similar to SVM classifier). Table 4 illustrates the results of the training error in ANFIS after 300 epochs.

According to Tables 3 and 4, the performance of SVM classifier was better than that of ANFIS classifier. Therefore, SVM classifier can better separate the two classes compared to the other classifier.

***TTCT Marks***

After the two groups of high and low marks were given to the SVM classifier, the accuracy for this class with linear features on ECG in task 1 was 96.86%, 95.48% in task 2, and 98.41% in task 3. However, it was 77.77% in task 1 of TTCT, 78.42% in task 2, and 80.88% in task 3, with nonlinear features on ECG. Error percentage of SVM network at best was observed for task 3, which was equal to 0.82%; and it was seen in the same task with ANFIS classifier, which was equal to 2.72%.

## Discussion

For the first time, creative thinking effects on ECG signals with some set of linear and nonlinear features were studied. Applying Wilcoxon statistical test, significant differences were observed between characteristics of rest and each of the three tasks of TTCT test, while this test did not reveal a significant difference between the second and the third stages. Wilcoxon for elaboration in task 3 and originality in tasks 1 and 2 showed a significant difference in the linear and nonlinear features on ECG signals.

Then to achieve higher accuracy percentage, two intelligent classifiers (ANFIS and SVM) were applied on the data.

Results revealed that SVM outperformed the proposed ANFIS model in classifying the ECG signals during the creativity states.

The SVM is a strong classifier, as well as it achieved the highest accuracy (99.63%) for linear features (time domain such as mean, RMSD, SDNN, median, maximum, minimum, and frequency domain such as ULF, VLF, LF, and HF) between rest and task 1 of TTCT.

Earlier, some research was done on heart rate (HR) and Electroencephalography (EEG) signals during TTCT (14). Moreover, some other studies were on functional Magnetic Resonance Imaging (fMRI) and Single-Photon Emission Computed Tomography (SPECT) during performing creativity tests (24).

These researches showed that power of alpha band in EEG signal and evidence from fMRI and SPECT, which showed activation in the frontal lobe of creative people and those with lower levels of creativity, is clearly distinguishable.

**Figure1:**
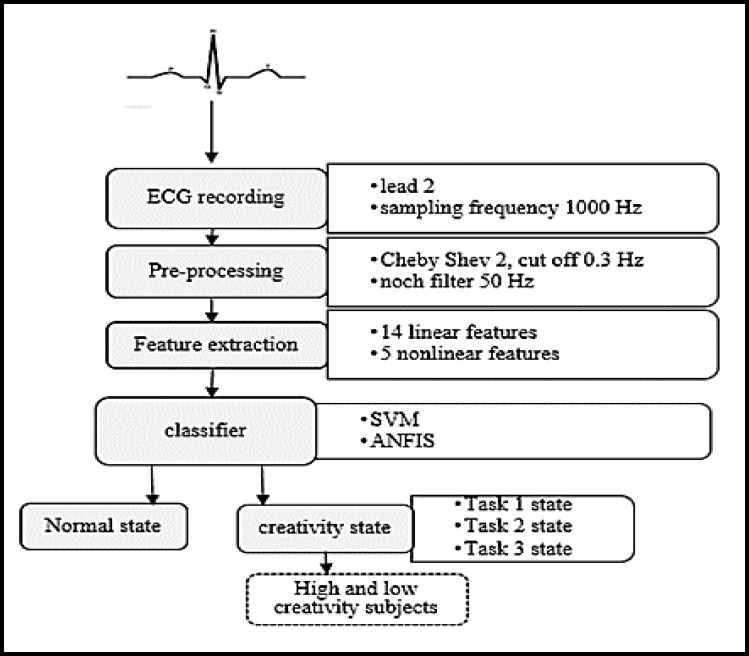
The Block Diagram of Classification Process Dashes Block Was Just Applied on SVM Output

**Figure2:**
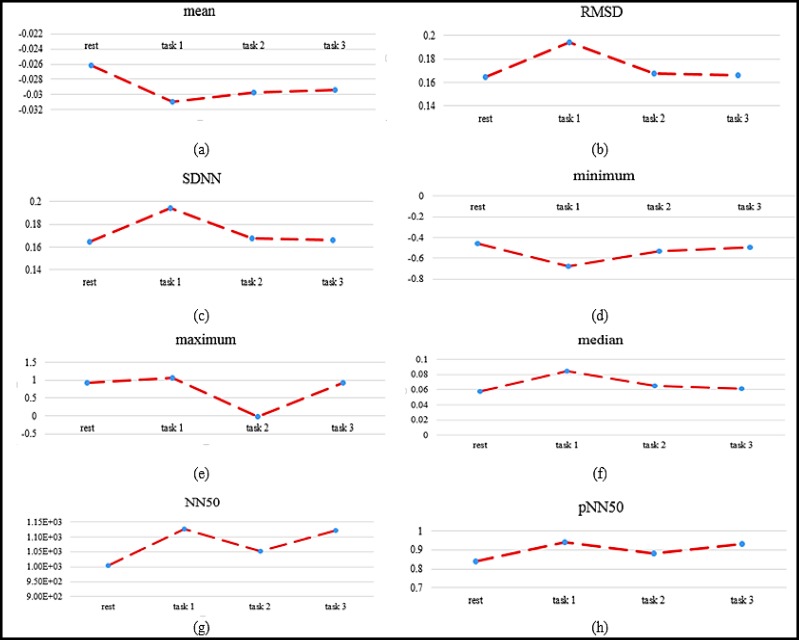
Average of Time Domain Parameters in Four Different States: (a) Average of Mean ECG Signal, (b) Root Mean Square Deviation, (c) Standard Deviation of the NN Interval, (d) Minimum, (e) Maximum, (f) NN Interval More 50 -Second, (h) Number of NN Interval More 50-Second

Mainly, analysis of ECG focused on the diagnosis of heart status (3). They could show cognitive behavior like stress on ECG signals (5-7). Some of the papers used similar methods (linear and nonlinear features on HR and ECG signals) but to detect disease and arrhythmia (25).

Previously, the SVM has been successfully used to classify biological data. The average accuracy of SVM classification has been 99.34% for discrimination arrhythmia (26) and 99.70% for the automatic detection of ECG waves (27). In addition, SVM classifier has been used in identifying the relationship between mental stress levels and biomedical signals, where its sensitivity and specificity was 89% (28).

***Future Discussion***

For future works, we intent to use the ECG signal with other biological signals like EEG to improve the results. In addition, larger sample size and use of other intelligent classifiers and networks may change or even improve our results.

## Conclusion

In this study, the sensitivity of 97.94% and specificity of 98.79% was achieved by SVM in combination with linear features in creativity states. Totally, the average accuracy of 98.42% was realized with linear features and 72.28% with nonlinear features on ECG signals during the creativity test.

## Limitations

All of These research was done on 52 engineering students. This statistical society is one of the limitations. If we manipulate a thoroughly data collection, the results would be extended on other subjects. Another limitation is age range. Selection of other age range can be useful in this study reports. 

**Figure3:**
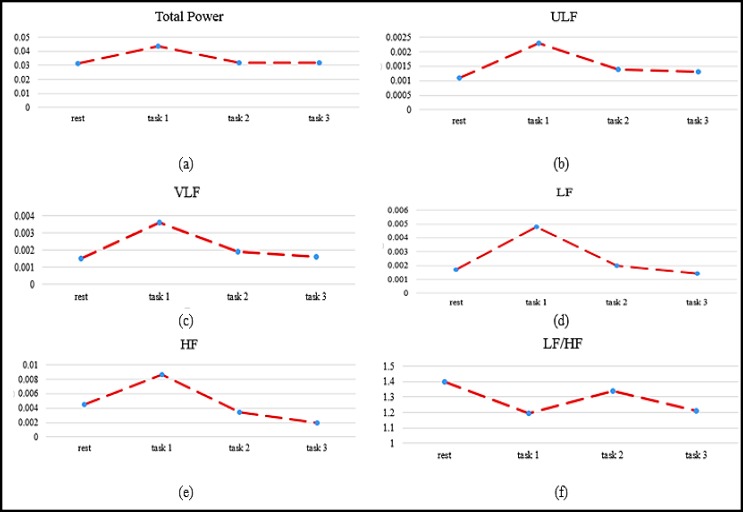
Average of Frequency Domain Parameters in Four Different States: (a) Average of Total Power of ECG, (b) Ultra-low Frequency Component, (c) Very Low Frequency Component, (d) Low Frequency Component, (e) High Frequency Component, (f) Ratio LF to HF

**Figure4:**
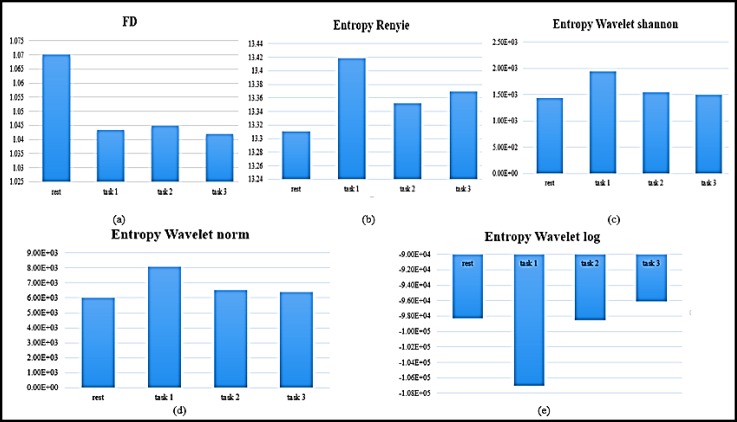
Nonlinear Features Changes in Four Different Stages: (a) Fractal Dimension, (b) Entropy Renyi, (c) Entropy Wavelet Shannon, (d) Entropy Wavelet Norm, (e) Entropy Wavelet Log
